# The Imbalance between Foxp3^+^Tregs and Th1/Th17/Th22 Cells in Patients with Newly Diagnosed Autoimmune Hepatitis

**DOI:** 10.1155/2018/3753081

**Published:** 2018-06-27

**Authors:** Ma Liang, Zhang Liwen, Zhuang Yun, Ding Yanbo, Chen Jianping

**Affiliations:** ^1^Department of Digestive Disease, The First People's Hospital of Changzhou, The Third Affiliated Hospital of Soochow University, Changzhou, Jiangsu, China; ^2^Department of Pediatrics, The Second People's Hospital of Changzhou, Affiliate Hospital of Nanjing Medical University, Changzhou, Jiangsu, China

## Abstract

This study is aimed at examining the potential role of regulatory T- (Treg-) Th1-Th17-Th22 cells in the pathogenic process of autoimmune hepatitis (AIH). The numbers of Foxp3^+^Tregs and Th1, Th17, and Th22 cells were measured in 32 AIH patients using flow cytometry. Moreover, a murine model of experimental autoimmune hepatitis (EAH) was also established and used to investigate the function of Treg-Th1-Th17-Th22 cells in disease progression. AIH patients undergoing an active state had significantly decreased numbers of CD3^+^CD4^+^CD25^+^Foxp3^+^Tregs and increased numbers of CD3^+^CD4^+^CD25^−^Foxp3^+^T, CD3^+^CD4^+^IFN-*γ*^+^Th1, CD3^+^CD4^+^IL-17^+^Th17, and CD3^+^CD4^+^IL-2^+^Th22 cells as well as higher levels of Th1/Th17/Th22-type cytokines compared to AIH patients in remission and HC. Additionally, the numbers of CD3^+^CD4^+^CD25^+^Foxp3^+^Tregs were negatively correlated with the numbers of Th1-Th17-Th22 cells. Also, the serum levels of IL-17A and IL-22 were correlated positively with liver injury (ALT/AST), whereas the serum levels of IL-10 were correlated negatively with hypergammaglobulinaemia (IgG, IgM) in AIH patients. Interestingly, the percentages of spleen Tregs, expression of Foxp3 mRNA, and liver IL-10 levels decreased, whereas the percentages of spleen Th1-Th17-Th22 cells, expression of T-bet/AHR/ROR*γ*t mRNA, and liver IFN-*γ*, IL-17, and IL-22 levels increased in the murine model of EAH. Our findings demonstrated that an imbalance between Tregs and Th1-Th17-Th22 cells might contribute to the pathogenic process of AIH.

## 1. Introduction

Autoimmune hepatitis (AIH) is a type of autoimmune inflammatory liver disease characterized by hypergammaglobulinaemia, circulating autoantibodies, and the liver injury with elevated levels of serum liver enzymes [1]. While genetic and environmental factors contribute to the pathogenic process of AIH [2], dysfunctional immune responses are also crucial for the development and progression of AIH [3]. However, the precise process of autoimmune responses remains obscure. Furthermore, although most patients respond well to immunosuppressive therapy, progression and end-stage liver disease occur in 10%–20% of cases and liver transplantation may be necessary [4]. Hence, further illustration of the mechanism underlying the breakdown of self-tolerance and leading to the development of AIH is of great importance in the management of patients with AIH.

It is generally believed that autoreactive T cells play a central role in the initiation and progression of AIH [5]. For example, numerous studies have revealed a transient and moderate increase in the production of Th1 cytokines, including interleukin-2 (IL-2), interferon-*γ* (IFN-*γ*), and tumor necrosis factor *α* (TNF-*α*) in AIH patients [6, 7]. In addition, recent studies also suggested that Th17 cells and further released cytokine IL-17 may induce accumulation of proinflammatory cells [8, 9], which contributed to the occurrence of AIH. In addition to well-characterized Th1 and Th17 lymphocytes, a new IL-22-producing T cell, termed Th22 cell, has been described as expressing its key cytokine IL-22, which can activate signal transduction and transcription 3 (STAT3) [10]. Recent studies have reported that the Th22 cell has hepatoprotective and antifibrotic functions in acute liver injury models of HBV [11]. Furthermore, Th22 cells are associated with inflammatory conditions and numerous autoimmune diseases, such as rheumatoid arthritis (RA) and systemic lupus erythematosus (SLE) [12, 13]. However, little is known about the role of Th22 cells in the pathogenesis of AIH. We believe that Th22 cells may partially contribute to the pathogenesis of AIH, and their expressions are likely time-dependent.

Regulatory T cells (Tregs) are important regulators of immune tolerance to self-antigens [14, 15], and their impairment has been associated with the development of autoimmune diseases [16, 17]. Human Tregs are CD4^+^CD25^+^, and their development and function depend on forkhead box protein 3 (FoxP3) expression [14, 15]. Previous studies in animal models have shown that a decreased number of CD4^+^CD25^+^FoxP3^+^T cells was detected [18], and function impairment was associated with the inflammatory activity of the liver [18, 19]. This reason is possible in that Tregs can actively suppress proinflammatory T cell responses [20]. At present, it is clear that Tregs can suppress Th1 and Th17 cell-mediated responses [20–22]. However, the relationship between Tregs and Th22 cells remains unclear. Moreover, recent studies have reported that FoxP3 is also expressed in some CD25^−^ T cells, which may be induced peripherally by environmental antigens [23–25]. While some studies have shown that CD4^+^CD25^−^FoxP3^+^T cells lacked inhibitory function [24], others indicated that CD4^+^CD25^−^FoxP3^+^T cells inhibited inflammation [25]. However, little is known about the expression of CD4^+^CD25^−^FoxP3^+^T cells in AIH patients and whether or not these cells are associated with disease development.

In the current study, we examined the numbers of circulating Foxp3^+^Tregs and Th1-Th17-Th22 cells, and the concentration of serum inflammatory cytokines in AIH patients, and used the mouse model of EAH to investigate the biological importance of Tregs and Th1/Th17/Th22 cells in the pathogenesis of AIH. The results of this experiment indicated a decreased Treg level and increased Th1, Th17, and Th22 cell levels, and all these cells contributed to the development of AIH. More importantly, a significantly negative correlation between Tregs and Th22 cells was noted in our experiment. These data indicated that Tregs and Th22 cells, in addition to classically described Th1 and Th17 cells, are involved in the process of AIH.

## 2. Material and Methods

### 2.1. Subjects

A total of 32 patients with newly onset AIH were recruited sequentially at the inpatient clinic of the Department of Digestive Disease, the First People's Hospital of Changzhou, the Third Affiliated Hospital of Soochow University, China. All patients were analyzed in an active disease state, as defined by ALT values or AST values above 50 U/mL. 23 patients were treated with prednisolone alone at a median dose of 100 mg at the time of acute presentation. 9 patients received prednisolone in combination with azathioprine at a median dose of 100 mg. Another 20 age-, gender-, and ethnicity-matched healthy controls (HCs) were recruited from the Department of Medical Examination Center of our hospital during the same period, and they had no history of any chronic inflammatory disease. Individual patients with AIH were diagnosed, according to the international criteria for the definitive diagnosis of AIH type I [4]. Patients with any other autoimmune diseases, connective tissue diseases (CTDs), chronic inflammatory diseases, recent infection, or a history of hepatitis virus infection or those who had received immunosuppressive therapies or glucocorticoid therapies within the past 6 months were excluded from the study. All subjects signed an informed consent form prior to the initiation of the study, and the study was approved by the Ethical Committee of the First People's Hospital of Changzhou.

### 2.2. Clinical Examination

The clinical data of each subject was collected from the hospital records. These data included age, sex, and laboratory tests. Individual subjects were subjected to routine laboratory tests for full blood cell counts and the concentrations of serum alanine transaminase (ALT) and aspartate aminotransferase (AST), antinuclear and smooth muscle antibody (ANA/SMA), IgG, IgM, and IgA by a biochemistry automatic analyzer (Roche Diagnostics, Branchburg, USA) and scattered turbidimetry on a Siemens special protein analysis instrument (Siemens Healthcare Diagnostics Products GmbH, Germany).

### 2.3. Animals

Adult C57BL/6 female mice were purchased from Nanjing Medical University (Jiangsu, China), and they were housed at controlled temperature with light-dark cycles, with free access to food and water. The experiments had been approved by the animal experimentation committee, and guidelines for human care for laboratory animals were observed. Each experiment was performed with five animals per group and repeated at least three times.

### 2.4. Induction of Experimental Autoimmune Hepatitis (EAH)

Induction of experimental autoimmune hepatitis (EAH) was performed through intraperitoneal injection of the mice with freshly prepared S-100 antigen at a dose of 0.5 to 2 mg/mL in 0.5 mL phosphate-buffered saline (PBS) emulsified in an equal volume of complete Freund's adjuvant (CFA) on day 0. A poster dose was given on day 7 as well [26]. Disease severity was assessed histologically on day 28 at the peak of disease activity. Disease severity was graded on a scale of 0 to 3 by an observer blinded to the identity of the samples.

### 2.5. Histological Evaluation

Liver tissues from sacrificed animals were fixed with 4% (*v*/*v*) PFA, dehydrated through a graded series of sucrose, frozen in an optimal cutting temperature (OCT) compound (Tissue TCK, Miles Elkhart, IN, USA), and stored at −80°C. 5 *μ*m cryostat sections were stained with hematoxylin and eosin (HE) to reveal and estimate the degree of inflammatory cell infiltration. Liver histology of EAH mice was scored using light microscopy and a modified Scheuer scoring scale, assigning scores for lobular inflammation (0, none; 1, mild-scattered foci of lobular-infiltrating lymphocytes; 2, moderate-numerous foci of lobular-infiltrating lymphocytes; and 3, severe-extensive pan–lobular-infiltrating lymphocytes).

### 2.6. Flow Cytometric Analysis of Intracellular Cytokine Staining

Freshly isolated peripheral blood mononuclear cells (PBMCs) of AIH patients and spleen mononuclear cells (SMNCs) of EAH mice were stimulated in duplicate with 50 ng/mL of phorbol myristate acetate (PMA) and 1.0 mg/mL of ionomycin (Sigma, St. Louis, MO, USA) in RPMI 1640 medium at room temperature in a humidified incubator with 95% air and 5% carbon dioxide for 2 hours and then cultured for another 4 hours in the presence of 0.5 mg/mL of brefeldin A (BFA, Sigma). The numbers of CD3^+^CD4^+^IFN-*γ*^+^Th1, CD3^+^CD4^+^IL-17^+^Th17, CD3^+^CD4^+^IL-22^+^Th22, and CD3^+^CD4^+^CD25^+^Foxp3^+^Tregs in individual samples and the frequency of CD3^+^CD8^−^IFN*γ*^+^Th1, CD3^+^CD8^−^IL17^+^Th17, CD3^+^CD8^−^IL22^+^Th22, and CD4^+^CD25^+^FoxP3^+^Tregs in EAH mice were determined by flow cytometry following intracellular staining with anticytokine antibodies.

### 2.7. Enzyme-Linked Immunosorbent Assay for IL-10, IFN-*γ*, IL-17, and IL-22

The cytokine levels in the serum (human) and in the supernatant of normalized hepatic tissue weight (mice) were collected and allowed to clot at 4°C for 2 hours. The clots were removed by centrifuging at 7000 rpm for 30 min in a refrigerated centrifuge. Serum/supernatant was obtained from all subjects by centrifugation and stored at −80°C for determination of cytokine levels. The levels of cytokines in the serum/supernatant were measured by enzyme-linked immunosorbent assay (ELISA) using commercially available IL-10, IFN-*γ*, IL-17, and IL-22 ELISA kits (Boster, Wuhan, China) according to the manufacturer's instructions.

### 2.8. RT-PCR

Total RNA was acquired from the hepatic tissue at each time point from EAH mice. The expressions of transcriptional repressors Foxp3, T-bet, ROR*γ*t, and AHR were then measured through real-time quantitative RT-PCR. Total RNA was extracted from hepatic tissue using the acid guanidinium thiocyanate-phenol-chloroform method. Total RNA of 2 *μ*g was reverse transcribed with hexamer and M-MuLV reverse transcriptase (New England Biolabs, Ipswich, MA) according to the manufacturer's instructions. cDNA corresponding to 25 ng of total RNA was subjected to quantitative PCR (qPCR) using primer sets and TaqMan probes corresponding to murine Foxp3, T-bet, AHR, ROR*γ*t, and GAPDH with qPCR Master Mix. All estimated mRNA values were normalized to GAPDH mRNA levels. The sizes of the PCR amplicons were as follows: Foxp3, 172 bp; T-bet, 86 bp; ROR*γ*t, 112 bp; and AHR, 98 bp. Promoter-specific primers were used for amplification: FOXP3 (forward: *5*′*-AGGAGAAAGCGGATACCA-*′*3* and reverse: *5*′*-TGTGAGGACTACCGAGCC-*′*3*), T-bet (forward: *5*′*-TCTTACTGGCTGGGAACACC-*′*3* and reverse: *5*′*-GCAGAGGGTAGGAATGTGGG-*′*3*), AHR (forward: *5*′*-AGAATCCCACATCCGCATGATT-*′*3* and reverse: *5*′*-CATTGGACTGGACCCACCTC-*′*3*), ROR*γ*t (forward: *5*′*-CCAGCTACCAGAGGAAGTCAA-*′*3* and reverse: *5*′*-TCCATGAAGCCTGAAAGCCG-*′*3*), and GAPDH forward: *5*′*-TGTGTCCGTCGTGGATCTGA-*′*3* and reverse: *5*′*-TTGCTGTTGAAGTCGCAGGAG-*′*3*).

### 2.9. Statistical Analysis

The differences between the groups were analyzed by Mann–Whitney *U* test and Wilcoxon signed-rank test using SPSS 18.0 software for unpaired and paired comparisons, respectively. The relationship between variables was evaluated using the Spearman rank correlation test. A two-sided *p* value < 0.05 was considered statistically significant.

## 3. Results

### 3.1. Demographic and Clinical Characteristics of Study Subjects

The baseline (before treatment) demographic characteristics of AIH patients and HC are summarized in Table 1. As expected, the concentrations of serum ALT, AST, *γ*-GGT, and ALP in the patients were significantly higher than those in the HC. Furthermore, 23 of 32 patients with new-onset AIH were positive for anti-ANA antibodies, while two were positive for anti-SMA antibodies. In addition, abnormally higher levels of serum IgG, IgM, and IgA were detected in the patients, as compared with those in the HC in this population. Furthermore, we analyzed the values of clinical parameters and in 19 AIH patients 8 weeks posttreatment. We found that the serum levels of ALT, AST, *γ*-GGT, and ALP were significantly descending compared to per-treatment values. Similarly, the titers IgG, IgM, and IgA were significantly decreased compared to the pretreatment levels (Table 2).

### 3.2. Altered Numbers of CD3^+^CD4^+^FoxP3^+^T Cells and Changed Levels of Serum IL-10 in AIH Patients

**Figure 1:**
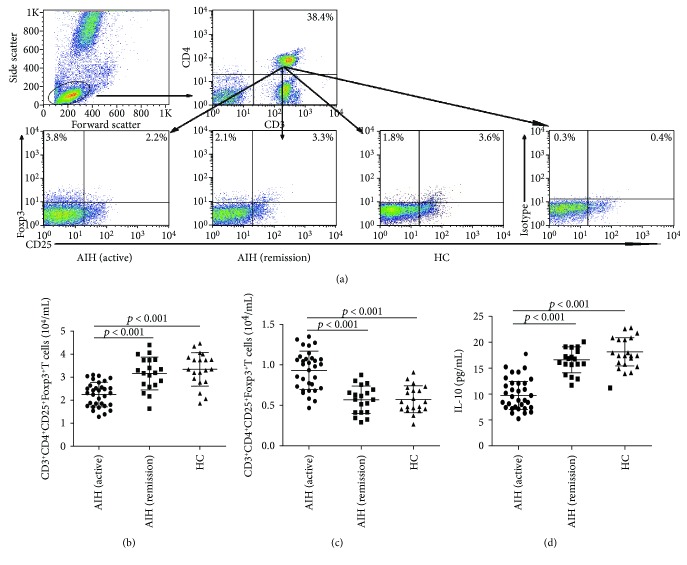
FACS analysis of the numbers of different subsets of circulating CD3^+^CD4^+^T cells and ELISA analysis of serum IL-10 in AIH patients. PBMCs were isolated from individual subjects, and PBMCs 5∗10^5^/tube were stained in duplicate with FITC-anti-CD3, PE-Cy7-anti-CD25, and PerCP-anti-CD4 or isotype controls, fixed, and permeabilized, followed by intracellular staining with PE-anti-Foxp3. The frequency of CD3^+^CD4^+^CD25^−^Foxp3^+^ and CD3^+^CD4^+^ CD25^+^Foxp3^+^T cells was determined by flow cytometry analysis. The cells were gated on living lymphocytes and then gated on CD3^+^CD4^+^ cells, and at least about 30,000 events were analyzed for each sample. The numbers of each type of CD3^+^CD4^+^Foxp3^+^T cells were calculated, according to the total numbers of PBMCs and the frequency of different types of CD3^+^CD4^+^Foxp3^+^T cells. The concentrations of serum IL-10 in individual subjects were determined by ELISA. (a) Flow cytometry analysis; (b) the numbers of CD3^+^CD4^+^CD25^+^Foxp3^+^T cells; (c) the numbers of CD3^+^CD^4+^CD25^−^Foxp3^+^T cells; (d) serum levels of IL-10. Data shown are representative FACS charts or the mean numbers of each type of cells per mL of peripheral blood and the mean levels of serum IL-10 in individual subjects from two separate experiments. The horizontal lines indicate the median values for each group. Data shown are representative charts of different subsets of CD3^+^CD4^+^T cells and serum IL-10 from individual groups of subjects (*n* = 20 for the HC, *n* = 32 for the patients at 0 week, and *n* = 19 for the patients at 8 weeks posttreatment).

We first characterized the levels of different subsets of CD3^+^CD4^+^FoxP3^+^T cells in PB of HC and AIH patients by flow cytometry analysis. As shown in Figure 1, AIH patients undergoing an active state had significantly decreased numbers of CD3^+^CD4^+^CD25^+^Foxp3^+^Tregs and increased numbers of CD3^+^CD4^+^CD25^−^Foxp3^+^T cells, compared to AIH patients in remission and HC. However, we did not find a significant difference in the numbers of CD3^+^CD4^+^CD25^+^Foxp3^+^Tregs and CD3^+^CD4^+^CD25^−^Foxp3^+^T cells between HC and AIH patients in remission.

Then, we further analyzed the levels of IL-10 in the serum of AIH patients and found a lower level of serum IL-10 in AIH patients undergoing an active state, compared to AIH patients in remission and HC (Figure 1(d)). However, we did not find a significant difference in the level of serum IL-10 between HC and AIH patients in remission. In addition, the concentrations of serum IL-10 were correlated positively with the numbers of CD4^+^CD25^+^Foxp3^+^T cells (*r* = 0.517, *p* = 0.002), but not with the numbers of CD4^+^CD25^−^Foxp3^+^T cells (*r* = 0.381, *p* = 0.511) in the patients.

### 3.3. Increased Numbers of Th1/Th17/Th22 Cells and Related Cytokines in AIH Patients

**Figure 2:**
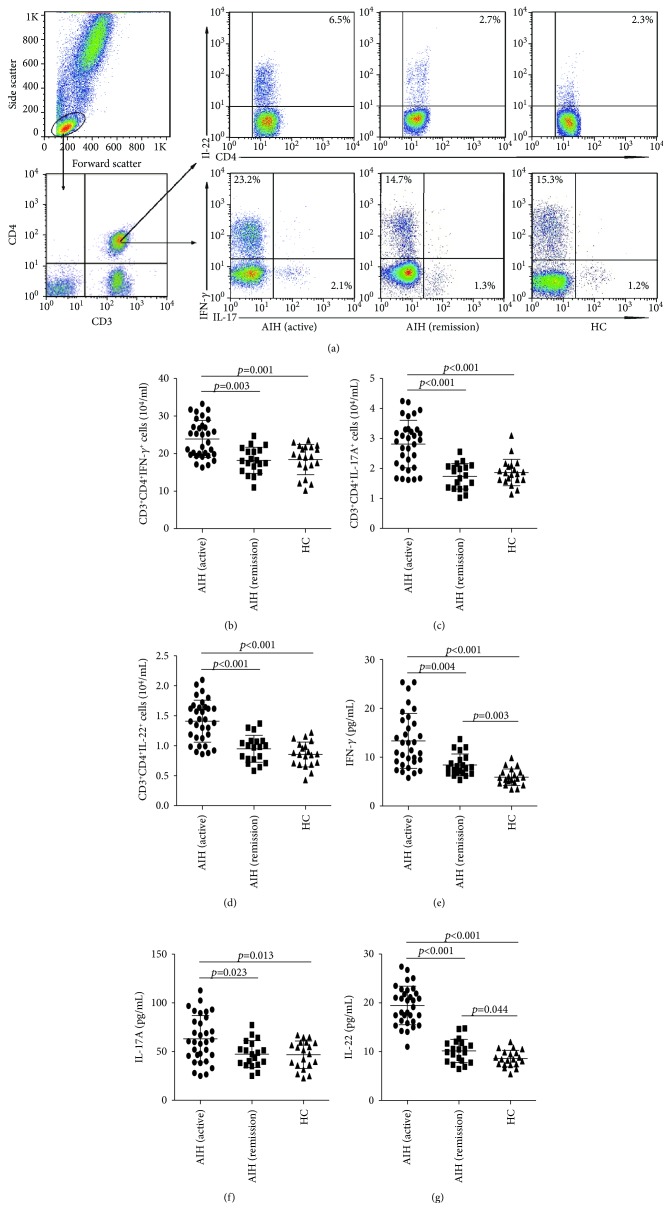
FACS analysis of the numbers of different subsets of circulating effector CD3^+^CD4^+^T cells and ELISA analysis of serum IFN-*γ*, IL-17, and IL-22 in AIH patients. PBMCs were isolated from individual subjects, and PBMCs 5∗10^5^/tube were stained in duplicate with APC-anti-CD4 and PerCP-anti-CD3 or isotype controls, fixed, and permeabilized, followed by intracellular staining with FITC-anti-IL-17 and PE-Cy7-anti-IFN-*γ* and PE-anti-IL-22. The frequency of CD3^+^CD4^+^IFN-*γ*^+^Th1, CD3^+^CD4^+^IL-17^+^Th17, and CD3^+^CD4^+^IL-22^+^Th22 cells was determined by flow cytometry analysis. The cells were gated on living lymphocytes and then gated on CD3^+^CD4^+^ cells, and at least about 30,000 events were analyzed for each sample. The numbers of each type of CD3^+^CD4^+^T cells were calculated, according to the total numbers of PBMCs and different types of CD3^+^CD4^+^T cells. (a) The flow cytometry analysis; (b–d) the numbers of CD3^+^CD4^+^IFN-*γ*^+^Th1, CD3^+^CD4^+^IL-17^+^Th17, and CD3^+^CD4^+^IL-22^+^Th22 cells; (e–g) serum levels of IFN-*γ*, IL-17, and IL-22. Data shown are representative FACS charts or the mean numbers of each type of cells per mL of peripheral blood in individual subjects from two separate experiments. The horizontal lines indicate the median values for each group. Data shown are representative charts of different subsets of CD3^+^CD4^+^T cells and serum IL-10 from individual groups of subjects (*n* = 20 for the HC, *n* = 32 for the patients at 0 week, and *n* = 19 for the patients at 8 weeks posttreatment).

Further comparison of different types of effector CD3^+^CD4^+^T cells found that AIH patients undergoing an active state had significantly increased numbers of CD3^+^CD4^+^IFN-*γ*^+^Th1, CD3^+^CD4^+^IL-17^+^Th17, and CD3^+^CD4^+^IL-22^+^Th22 cells and higher levels of serum Th1 cytokine IFN-*γ*, Th17 cytokine IL-17A, and Th22 cytokine IL-22, compared to AIH patients in remission and HC (Figure 2). However, we did not find a significant difference in the numbers of Th1/Th17/Th22 cells and their related cytokines between HC and AIH patients in remission.

### 3.4. Negative Correlation between Tregs and Th1/Th17/Th22 Cells in AIH Patients

**Figure 3:**
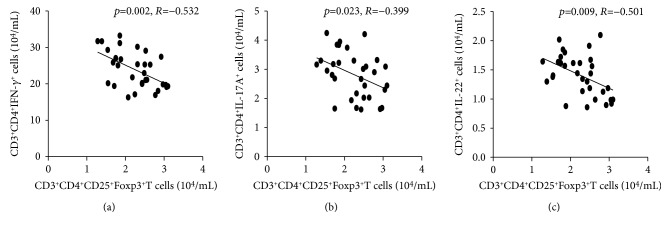
Correlation among the numbers of different subsets of circulating CD3^+^CD4^+^T cells in AIH patients. Correlation between the numbers of CD3^+^CD4^+^CD25^+^Foxp3^+^T cells and the numbers of CD3^+^CD4^+^IFN-*γ*^+^Th1 (a), CD3^+^CD4^+^IL-17^+^ Th17 (b), and CD3^+^CD4^+^IL-22^+^Th22 cells (c) in AIH patients.

Tregs can suppress the proliferation and activation of other effector CD3^+^CD4^+^T cells [20–22]. In order to better characterize the role of Tregs in Th1/Th17/Th22 cells, correlation analysis was performed, and the results showed that decreased numbers of Tregs were significantly negatively correlated with the numbers of Th1 and Th17 cells in AIH patients undergoing an active state (Figures 3(a) and 3(b)). In addition, the numbers of Tregs also had a negative correlation with peripheral Th22 cell level with statistical significance in AIH patients undergoing an active state (Figure 3(c)). Moreover, further analysis indicated that decreased numbers of Tregs were negatively correlated with the concentrations of serum IFN-*γ*, IL-17, and IL-22 in AIH patients undergoing an active state (data not shown). However, we did not observe any significant correlation among these cells in AIH patients in remission or HC. These data suggested that Tregs might also regulate Th22 cell-mediated responses, in addition to classically described Th1 and Th17 cells.

### 3.5. The Relationship between CD3^+^CD4^+^T Cell-Related Cytokines and Clinical Parameters in AIH Patients

**Figure 4:**
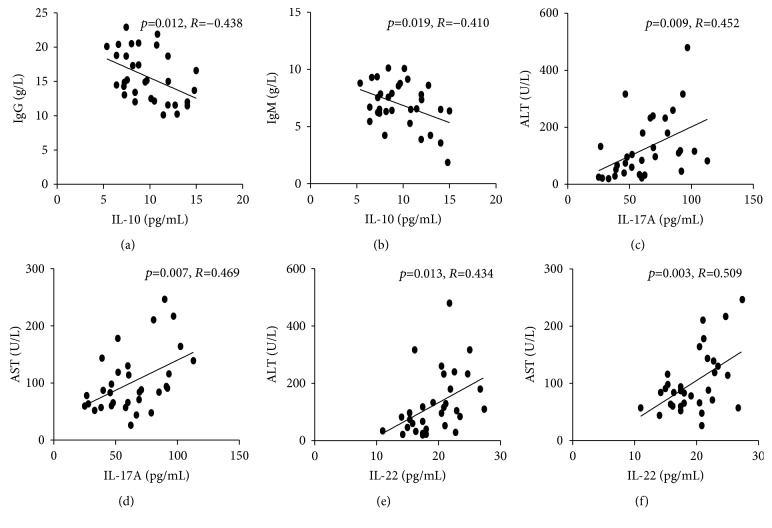
Correlation between serum levels of CD3^+^CD4^+^T cell-related cytokines and the values of clinical parameters in AIH patients. (a, b) Correlation between serum levels of IL-10 and titer IgG/IgM in AIH patients; (c, d) correlation between serum levels of IL-17A and ALT/AST in AIH patients; (e, f) correlation between serum levels of IL-22 and ALT/AST in AIH patients.

To understand the importance of Tregs-Th1-Th17-Th22 cells, we analyzed the potential association of the levels of these CD3^+^CD4^+^T cell-related cytokines with the values of clinical parameters in AIH patients undergoing an active state. We found that the concentrations of serum IL-10 were correlated negatively with the concentrations of serum IgG and IgM, whereas the concentrations of serum IL-17 and IL-22 were positively correlated with the levels of ALT and AST (Figure 4). However, there was no other significant correlation between the levels of these CD3^+^CD4^+^T cell-related cytokines with any of the clinical parameters tested in this population (data not shown).

### 3.6. Statistical Increase in Th1/Th17/Th22 Cells and Decrease in Tregs in EAH Mice

**Figure 5:**
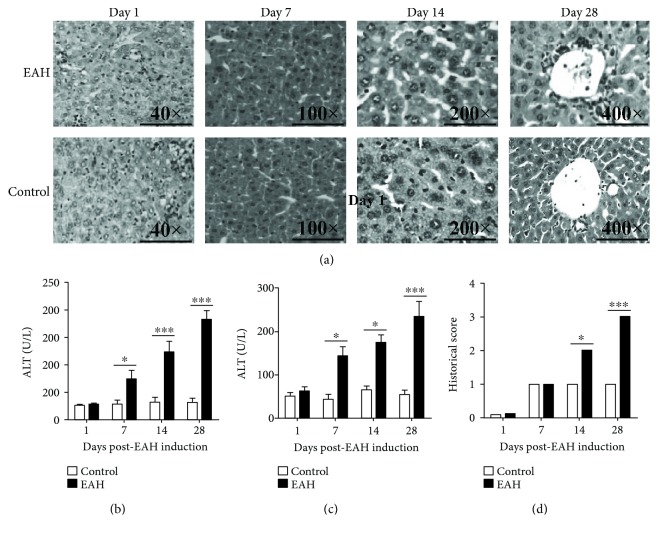
Biochemical and histological analyses in EAH mice. Ten mice (5 from the EAH group and 5 from the control group) were killed at each time point (7, 14, and 28 days). (a) Representative histological picture of liver lesions in animals after standard induction of EAH and controls (magnification, 40x, 100x, 200x, or 400x). (b) Serum ALT levels progressively upregulated from 1 to 28 days. (c) Serum AST levels progressively upregulated from 1 to 28 days. (d) Histological score of liver lesions in mice after standard induction of EAH. The horizontal lines indicate the mean values of the different groups. ^∗^*p* < 0.05 and ^∗∗∗^*p* < 0.01.

**Figure 6:**
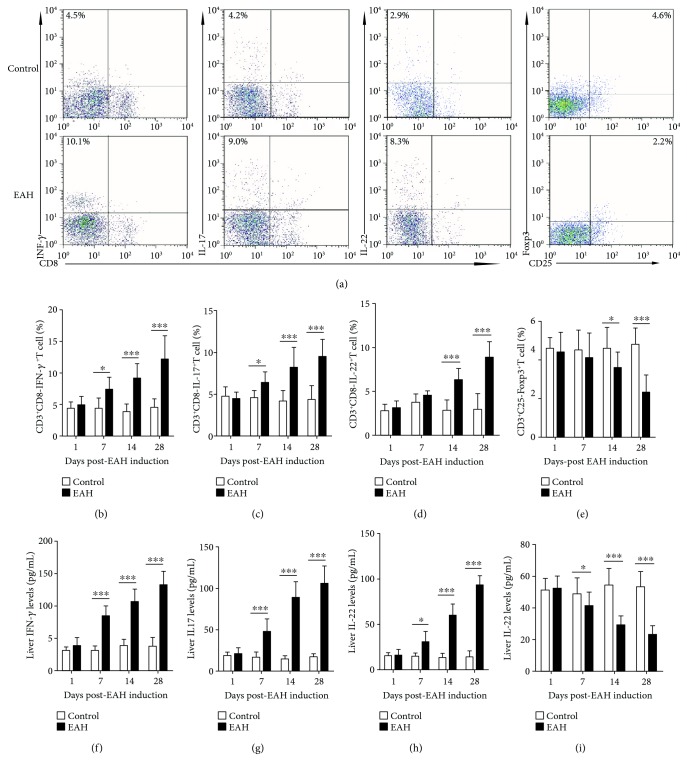
Flow cytometry of Treg/Th1/Th17/Th22 cells in SMNCs and ELISA analysis of hepatic levels of IL-10, IFN-*γ*, IL-17A, and IL-22 in EAH mice. Ten mice (5 from the EAH group and 5 from the control group) were killed at each time point (1, 7, 14, and 28 days). (a) Flow cytometry analysis of Tregs and Th1, Th17, and Th22 cells from the spleen on the 14th day. (b–e) Percentages of Tregs and Th1, Th17, and Th22 cells at each time point from EAH and control mice were analyzed by FACS. (f–i) Liver IL-10, IFN-*γ*, IL-17A, and IL-22 levels at each time point from EAH and control mice were used for ELISA. The horizontal lines indicate the mean values of the different groups. ^∗^*p* < 0.05 and ^∗∗∗^*p* < 0.01.

To understand the effects of regulatory and effector T cells in vivo, we had successfully established the murine model of EAH. Compared with the control group, EAH mice had obvious liver injury evidenced by liver edema with a rising liver index and dramatically enhanced serum levels of AST and ALT (Figure 5). Splenocytes were collected from mice at each time point, and flow cytometry was performed to analyze the percentages of IFN-*γ*-producing Th1 cells, IL-17-producing Th17 cells, IL-22-producing Th22 cells, and FoxP3^+^Tregs in EAH and control mice. We found that IFN-*γ*-producing CD3^+^CD8^−^ T cells (Th1 cells) and IL-17-producing CD3^+^CD8^−^ T cells (Th17 cells) in the experimental group significantly increased on the 7th, 14th, and 28th days of post-EAH induction, and IL-22-producing CD3^+^CD8^−^T cells (Th22 cells) obviously increased on the 14th and 28th days of post-EAH induction compared with those in the control group (Figures 6(a)–6(d)). Furthermore, our data also showed that the liver levels of IFN-*γ*, IL-17, and IL-22 on the 7th, 14th, and 28th days of post-EAH induction were statistically higher in the experimental group than in the control group (Figures 6(f)–6(h)). Moreover, CD3^+^CD4^+^CD25^+^FoxP3^+^Tregs and liver levels of IL-10 obviously decreased on the 7th, 14th, and 28th days of post-EAH induction compared with those in the control group (Figures 6(e) and 6(i)).

### 3.7. Marked Changes of Hallmark Transcription Factor of Treg/Th1/Th17/Th22 Cells in EAH Mice

**Figure 7:**
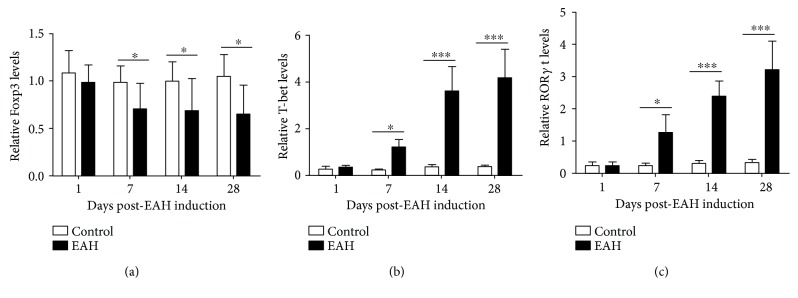
Foxp3, T-bet, ROR*γ*t, and AHR expressions in the hepatic tissue of EAH mice. Ten mice (5 from the EAH group and 5 from the control group) were killed at each time point (1, 7, 14, and 28 days). (a–d) The changes of Foxp3, T-bet, ROR*γ*t, and AHR expressions in the hepatic tissue of EAH mice, respectively. ^∗^*p* < 0.05 and ^∗∗∗^*p* < 0.001.

The transcriptional repressors FoxP3, T-bet, ROR*γ*t, and AHR were the hallmark transcription factors of Tregs, Th1 cells, Th17 cells, and Th22 cells, respectively. Immunohistochemistry and real-time PCR were employed to further ascertain in detail whether Tregs and Th1, Th17, and Th22 cells had the potential of local changes in the process of EAH induction. The results found that the mRNA expression levels of T-bet, ROR*γ*t, and AHR in experimental mice increased remarkably 7, 14, and 28 days post-EAH induction compared with those in the control group, whereas the messenger RNA (mRNA) encoding Foxp3 was expressed in different ways (Figure 7).

## 4. Discussion

Although dysregulated activation of effector CD4^+^T helpers (Th) has been associated with the pathogenic process of autoimmune hepatitis [3, 5–7], this specific mechanism is still contradictory. In this study, we found a strong Th1 and Th17 proinflammatory response with increased levels of serum/liver Th1-type (IFN-*γ*) and Th17-type (IL-17A) cytokines in AIH patients undergoing an active state and EAH mice, indicating that Th1 and Th17 cells have critical functions in the pathogenic process of AIH, which were consistent with previous studies [6–9]. The significantly changed Th1 and Th17 cells may stem from the inflammatory environment, which preferably activates naive helper T cells towards Th1 and Th17 directions. In addition to Th1 and Th17 cells, Th22 cells expressing aryl hydrocarbon receptor (AHR) play a critical role in the development of immune and inflammatory diseases by producing proinflammatory cytokine IL-22 [10–12, 27, 28]. However, the possible mechanisms of Th22 cells and IL-22 in the development of AIH remain unknown. Notably, our data showed also a high expression level of Th22 cells and serum/liver Th22 type (IL-22) in AIH patients undergoing an active state and EAH mice. On the other hand, consistent with changes in Th1 and Th17 cells, the levels of Th22 cells and IL-22 decreased significantly after immunosuppressive drug treatment. These results suggested that Th22 cells, like Th1 and Th17 cells, also have major functions in the pathogenesis of AIH. More importantly, the levels of Th17/Th22-type (IL-17A, IL-22) cytokines were correlated positively with the levels of serum ALT/AST, which are a hallmark of liver injury, suggesting that Th22 and Th17 cells contribute to the progression of liver damage and fibrosis [6, 7, 11, 28]. Furthermore, our data showed a sustained replication of Th1/Th17/Th22-related mRNA (T-bet, ROR*γ*t, and AHR) in a time-dependent manner in EAH mice, which might be the direct cause of the gradual elevation of Th1/Th17/Th22 cells [29–31].

In addition to effector T cells, Tregs are important regulators of immune tolerance and inflammation response. In this study, we found lower levels of Tregs in AIH patients and in EAH mice in a time-dependent manner, which were consistent with previous studies [18, 32, 33] but were different from another report [19]. Conflicting results may be due to differences in methodology, or detection markers of the definition of Tregs, because Moritz et al. considered CD4^+^CD25^high^CD127^low^FoxP3^+^T cells as Tregs in their study. Moreover, these inconsistencies are partly due to enrollment of patients regardless of the phase of their disease. More importantly, we found that the concentrations of serum IL-10 were significantly higher and correlated positively with the numbers of Tregs and correlated negatively with the levels of serum immunoglobulins in AIH patients undergoing an active state, which might interfere with plasma B cell differentiation and inhibit immunoglobulin production, contributing to the pathogenic process of AIH [34]. These results suggested that deficient Tregs might contribute to the breakdown in tolerance in the development of AIH. Recent studies have reported that CD4^+^CD25^−^T cells can be induced for FoxP3 expression [23]. Functionally, some studies showed that CD4^+^CD25^−^FoxP3^+^T cells have no inhibitory function; others indicated that these cells can inhibit inflammation in immune rejection and autoimmune diseases [24, 25]. In this study, our data showed increased numbers of CD3^+^CD4^+^CD25^−^FoxP3^+^T cells in AIH patients undergoing an active state. On the other hand, the numbers of CD3^+^CD4^+^CD25^−^FoxP3^+^T cells decreased after immunosuppressive drug treatment. However, there was no correlation between these cells and the clinical indicators. Hence, these new findings suggested that CD3^+^CD4^+^CD25^−^FoxP3^+^T cells may not be inhibitory Tregs. However, the role of these changes needs more studies to investigate.

Given that Tregs have the ability to inhibit the proliferation and activation of autoreactive Th1 and Th17 cells [20–22], we further set out to analyze the correlation between Tregs and Th1-Th17 cells in AIH patients. Our data demonstrated that Tregs were negatively correlated with Th1 and Th17 cells as well as with Th1-type (IFN-*γ*) and Th17-type (IL-17A) cytokines, which were consistent with previous research that showed suppression of Th1 cell-mediated and Th17 cell-mediated responses by Tregs through inhibition of monocyte-derived TGF-*β* or IL-10 or IL-6 [21, 22]. These results indicated that the imbalance of Treg-to-Th1 and Treg-to-Th17 ratios might favor the pathogenesis of AIH. Furthermore, we further examined the association between Tregs and Th22 cells and found that Tregs were also negatively correlated with Th22 cells and IL-22 in the circulating levels of AIH patients, suggesting that deficient Tregs also enhanced Th22-mediated immune responses. This process might be due to the secretion of particular cytokines by Tregs. However, the specific mechanisms of Treg-regulated Th22 cell activation and differentiation need to be further explored in the process of AIH.

In conclusion, our data showed significantly reduced numbers of Tregs and serum IL-10 levels and increased numbers of Th1, Th17, and Th22 cells as well as higher levels of Th1-Th17-Th22-type cytokines in AIH patients and EAH mice. Additionally, the numbers of Tregs were negatively correlated with the numbers of Th1-Th17-Th22 cells and levels of IFN-*γ*, IL-17A, and IL-22. More importantly, serum IL-17A and IL-22 levels were positively correlated with the liver damage, whereas serum IL-10 levels were negatively correlated with hypergammaglobulinaemia. These novel findings suggested that effector Th1/Th17/Th22-cell-mediated immune response might be controlled by Tregs and the imbalance between Tregs and the Th1/Th17/Th22 axis might contribute to the process of AIH. We recognized that our study had limitations, such as the lack of functional study of Treg-regulated Th22 cells in the pathogenic process of AIH. Therefore, further studies on the molecular mechanisms are needed further to be carried out.

## Figures and Tables

**Table 1:** The demographic and clinical characteristics of subjects.

Parameters	AIH	HC
Number	32	20
Age (years)	48 (37–76)	51 (41–74)
Gender: female/male	24/8	14/6
ALT (U/L)	125.9 ± 108.3^∗^	27.2 ± 8.2
AST (U/L)	101.1 ± 53.7^∗^	22.7 ± 5.7
*γ*-GT (U/L)	89.1 ± 30.3^∗^	25.1 ± 7.4
ALP (U/L)	133.4 ± 37.1^∗^	89.5 ± 23.6
Anti-ANA (+)	23/32 (71.8%)^∗^	0/20 (0%)
Anti-ANA titer	1 : 640 (1 : 80–1 : 10000)	—
Anti-SMA (+)	2/30 (6.25%)	0/20 (0%)
Anti-SMA titer	1 : 1000 (1 : 160–1 : 3200)	—
IgG (g/L)	15.9 ± 3.7^∗^	7.8 ± 2.3
IgM (g/L)	6.9 ± 1.9^∗^	2.64 ± 0.87
IgA (g/L)	4.07 ± 2.3^∗^	1.6 ± 1.1
WBC (×10^9^/L)	7.93 (5.6–11.2)^∗^	5.08 (3.9–9.2)

Data shown are the real case number or mean ± SD. Normal values: ALT: <40 IU/L; AST: <40 IU/L. Normal values: ANA: <1 : 80; SNA: <1 : 80. IgG (normal range: 7–16 g/L); IgM (normal range: 0.7–4.6 g/L); and IgA (normal range: 0.4–2.3 g/L). HC: healthy control; AIH: autoimmune hepatitis. ^∗^*p* < 0.05 versus HC.

**Table 2:** Effect of treatment on the values of clinical measures in follow-up AIH patients.

Parameters	Before treatment	After treatment
Number	19	19
Age (years)	42 (37–76)	42 (37–76)
Gender: female/male	16/3	16/3
ALT (U/L)	184.6 ± 105.3^∗^	37.2 ± 10.4
AST (U/L)	126.4 ± 54.8^∗^	43.6 ± 11.5
*γ*-GT (U/L)	105.6 ± 26.1^∗^	76.1 ± 24.9
ALP (U/L)	152.5 ± 33.3^∗^	93.7 ± 23.2
IgG (g/L)	17.7 ± 3.1^∗^	8.7 ± 2.9
IgM (g/L)	7.8 ± 1.6^∗^	4.5 ± 2.3
IgA (g/L)	3.9 ± 1.7^∗^	2.6 ± 1.1

Data shown are the real case number or mean ± SD. Normal values: ALT: <40 IU/L; AST: <40 IU/L. IgG (normal range: 7–16 g/L); IgM (normal range: 0.7–4.6 g/L); IgA (normal range: 0.4–2.3 g/L). HC: healthy control; AIH: autoimmune hepatitis. ^∗^*p* < 0.05 versus posttreatment.

## Data Availability

The data used to support the findings of this study are available from the corresponding author upon request.
